# Recent developments in cancer research: Expectations for a new remedy

**DOI:** 10.1002/ags3.12440

**Published:** 2021-02-15

**Authors:** Koji Ando, Qingjiang Hu, Yuta Kasagi, Eiji Oki, Masaki Mori

**Affiliations:** ^1^ Department of Surgery and Science Kyushu University Fukuoka Japan

**Keywords:** exosomes, immunotherapy, microbiome, organoid

## Abstract

Cancer research has made remarkable progress and new discoveries are beginning to be made. For example, the discovery of immune checkpoint inhibition mechanisms in cancer cells has led to the development of immune checkpoint inhibitors that have benefited many cancer patients. In this review, we will introduce and describe the latest novel areas of cancer research: exosomes, microbiome, immunotherapy. and organoids. Exosomes research will lead to further understanding of the mechanisms governing cancer proliferation, invasion, and metastasis, as well as the development of cancer detection and therapeutic methods. Microbiome are important in understanding the disease. Immunotherapy is the fourth treatment in cancer therapy. Organoid biology will further develop with a goal of translating the research into personalized therapy. These research areas may result in the creation of new cancer treatments in the future.

## Graphical abstract

Cancer research has made remarkable progress and new discoveries are beginning to be made. In this review, we will introduce and describe the latest novel areas of cancer research: exosomes, microbiomes, immunotherapy, and organoids.

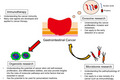

## 1 INTRODUCTION

The cancer research field has developed significantly through use of new equipment and technology. One example of new technology is Next‐Generation Sequencing (NGS). Also known as high‐throughput sequencing, NGS is the catch‐all term used to describe a number of different modern nucleic acid sequencing technologies. These methods allow for much quicker and cheaper sequencing of DNA and RNA compared with the previously used Sanger sequencing, and as such have revolutionized the study of genomics and molecular biology. NGS also allows for easier detection of mutations in cancer samples, leading to development of many new agents that can be used to treat patients. For example, if the *RAS* gene status is detected as wild type in a colorectal cancer patient, then an anti‐EGFR antibody, such as cetuximab or panitumumab, can be used for treatment.

A liquid biopsy, also known as fluid biopsy or fluid phase biopsy, is the sampling and analysis of non‐solid biological tissue, primarily blood.^1^ It is being used as a novel way to detect cancer. Like a traditional biopsy, this type of technique is mainly used as a diagnostic and monitoring tool for diseases, and also has the added benefit of being largely noninvasive. Therefore, liquid biopsies can be performed more frequently, allowing for better tracking of tumors and mutations over a duration of time. This technique may also be used to validate the effectiveness of a cancer treatment drug by taking multiple liquid biopsy samples in the span of a few weeks. It may also prove to be beneficial for monitoring relapse in patients after treatment.

Novel devices and drugs have also been developed and used for cancer treatment. For surgery procedures, robotic‐assisted laparoscopic surgery has evolved and made it possible to visualize the fine movement of the forceps in three dimensions. This method is now used in esophageal, gastric, and rectal cancer surgeries in Japan.^2^, ^3^, ^4^

Recently, immunotherapy became an additional method for treating cancer patients. The discovery of the immune checkpoint by Dr Honjo led to the development of immune checkpoint inhibitors.^5^ Despite these developments, gastrointestinal cancers are still a major problem in need of new treatment methods. In this review, we introduce and describe four new areas of cancer research that may contribute to cancer treatment in the future: exosomes, microbiome, immunotherapy, and organoids.

## 2 AN APPLICATION OF EXOSOME RESEARCH IN CANCER THERAPY

An exosome is a small particle that is secreted by cells. Its size can range from 50 to 150 nm and has a surface consisting of proteins and lipids that originate from the cell membrane. Additionally, proteins and nucleic acids, such as DNA, microRNAs, and mRNAs, can be found inside the exosome as its “cargo.”^6^ Recently, many researchers have discovered that exosomes are involved in the mechanisms of various diseases. As mentioned above, various functional compounds, such as microRNAs, mRNAs, and proteins, can be contained within exosomes.^7^, ^8^ Many cells use secretion of exosomes to communicate with one another, and these exosomes can even reach distant cells. Cancer cells can also secrete exosomes that contain molecules beneficial to cancer growth. For example, microRNAs found in cancer exosomes can modulate gene expression to induce angiogenesis in the tumor microenvironment, which supports metastasis.^9^ Exosomes released from cancer cells can also reportedly break the blood‐brain barrier, which makes it contribute to brain metastasis.^10^, ^11^ Cancer cells themselves are similarly affected by the exosomes secreted by the surrounding normal cells.^12^ In one case, the exosomes secreted by bone marrow‐delivered mesenchymal stem cells can force cancer cells into a dormant state.^13^ These dormant cancer cells become resistant to chemotherapy and are involved in long‐term disease recurrence. Thus, exosomes are deeply involved in cancer proliferation, invasion, and metastasis, as well as in the formation of the tumor microenvironment and pre‐metastatic niche.^13^ Further research on cancer‐related exosomes is ongoing.

Knowledge of exosomes can be applied to cancer treatment. If the secretion of exosomes from cancer cells can be prevented, then signal transduction supporting the formation of the tumor microenvironment and pre‐metastatic niche can be blocked. Work focusing on the removal of cancer exosomes is now ongoing.^14^

Exosomes can also be utilized for cancer diagnosis. Exosomes secreted by many cell types are found in various body fluids, such as blood and urine. Capturing and analyzing exosomes from cancer cells can be used to detect the presence of disease.^15^ Obtaining blood or urine from patients is not very invasive or painful. Since many molecules, such as various proteins, DNA, and microRNAs, can be found in exosomes from normal cells, it is important to distinguish them from cancer‐related ones. If exosomes are to be used for cancer diagnosis, then specific biomarkers need to be discovered. Additionally, the development of a method to detect these exosomes must be done. Currently, exosome detection methods for exosomes abundantly found in the serum of colorectal and pancreatic cancer patients, as well as exosomes found in the urine of bladder cancer patients, are being developed.^16^, ^17^ Thus, further understanding of the mechanisms governing cancer proliferation, invasion, and metastasis, as well as the development of cancer detection and therapeutic methods, is significantly affected by exosome research.

## 3 MICROBIOME IN CANCER RESEARCH

A large number of microorganisms inhabit the human body. These microorganisms include bacteria, viruses, and fungi. Among them, bacteria have the most important relationship with the human body. Bacteria can live anywhere within the human body, including the digestive tract, respiratory system, and oral cavity.^18^, ^19^, ^20^ In particular, bacteria in the digestive tract are rich in type and number,^21^ with possibly 1000 types and more than 100 trillion individual bacterial cells present.^22^, ^23^ The overall population of various bacteria found in the human intestine is referred to as the “intestinal flora.” Recently, the terms “microbiota” or “microbiome” have also been widely used.

Recent advancements with NGS have led to a much more precise understanding of the intestinal microbiome.^24^ The bacteria in the human microbiome mainly belong to four phyla: Firmicutes, Bacteroidetes, Proteobacteria, and Actinobacteri. Of these, Firmicutes and Bacteroidetes are the most dominant species. It is reported that microbiome vary depending on age and race.^25^, ^26^ Dysbiosis is a condition in which the diversity of the microbiome is reduced. Dysbiosis is reportedly involved in various diseases such as inflammatory bowel disease, colorectal cancer, obesity, diabetes, and allergic diseases.^27^, ^28^, ^29^ For example, bacteria such as *Atopobium parvulum* and *Actinomyces odontolyticus* increase in number during the early stages of colorectal cancer (adenomas or intramucosal cancers) and decrease in number during cancer progression.^30^ This suggests that a specific microbiome is associated with early stages of colorectal cancer development, which may be useful knowledge for early cancer detection.

Various studies have also been conducted to elucidate the relationship between the microbiome and the human immune system.^31^ The IgA antibody, which is one of the most important elements in the intestinal immune system, is believed to play a role in the elimination of pathogens and maintenance of the intestinal environment. The IgA antibody recognizes, eliminates, and neutralizes pathogenic bacteria and toxins. It also maintains a symbiotic relationship by recognizing and binding to the normal microbiome of the host.^32^ Mice lacking a microbiome have reduced production of the IgA antibody. A microbiome is required for IgA antibody differentiation. Recent studies have identified W27IgA antibodies that have the ability to bind to various bacteria.^33^ Oral administration of a W27IgA antibody to enteritis model mice suppressed enteritis by altering the microbiome. This W27IgA antibody can recognize a part of the amino acid sequence of serine hydroxymethyl transferase, which is a metabolic enzyme involved in bacterial growth. The W27IgA antibody can suppress the growth of *E coli* by binding to them. However, the W27IgA antibody does not bind to bacteria that suppress enteritis, such as *bifidobacteria* and lactic acid bacteria.^33^ Thus, the microbiome is deeply involved in human intestinal immunity. Recently, it is having been established that the microbiome is not only involved in intestinal immunity, but also in the systemic immune system.

As the analysis of the microbiome progresses, the pathophysiology of various diseases, such as cancers, and its relationship with the regulatory function of the human immune system will be further elucidated. It has been demonstrated that *F nucleatum* plays a role in the development and progression of colon adenomas and colorectal cancer. It is also related to lymph node metastases and distant metastasis.^34^, ^35^ Also, microbiome is associated with hepatocellular carcinoma.^36^ Studying microbiome will give us some clue in the development and remedy for gastrointestinal cancers (Table 1).

**TABLE 1:** Gastrointestinal cancer and their related microbiome

Gastrointestinal cancer	Related microbiome
Gastric cancer	*Helicobacter pylori*
Colorectal cancer	*Atopobium parvulum*
	*Fusobacterium nucleatum*
	*Actinomyces odontolyticus*
	*Solobacterium moorei*
	*Parvimonas micra*
	*Peptostreptococcus stomatis*
	*Desulfovibrio longreachensis*
	*Bacteroides fragiis*
	*Peptostreptococcus anaerobius*
	*Escherichia coli*
	*Campylobacter jejuni*
Hepato cellular carcinoma	*Bacteroides*
	*Lachnospiracea incertae sedis*
	*Clostridium XIVa*
	*Helicobacter hepaticus*
Biliary tract cancer	*Salmonella enterica*
	*Salmonella typhi*
	*Helicobacter hepaticus*
	*Helicobacter pylori*
	*Helicobacter bilis*
Pancreatic cancer	*Porphyromonas gingivalis*

## 4 THE RISE OF IMMUNOTHERAPY IN CANCER TREATMENT

For many years, surgery, chemotherapy, and radiation therapy were the main methods of cancer treatment. In addition to these therapies, immunotherapy has recently attracted great attention worldwide (Table 2).^37^, ^38^ Under normal circumstances, a cancer antigen will activate the patient's immune system to attack the cancer cells. However, sometimes the immune system does not recognize the cancer cells as non‐self, or it simply fails to attack them. This can result in the development and progression of cancer.

**TABLE 2:** Immune checkpoint inhibitors

Immune checkpoint inhibitor	Target molecule	Target cancer
Ipilimumab	CTLA‐4	Malignant melanoma, Renal cell carcinoma, (combination with nivolumab) MSI‐H CRC
Tremelimumab	CTLA‐4	(combination with Durvalumab) Non‐small cell lung cancer, Head and neck cancer
Pembrolizumab	PD‐1	Malignant melanoma, Non‐small cell lung cancer, MSI‐H solid tumors
Nivolumab	PD‐1	Malignant melanoma, Non‐small cell lung cancer, Head and neck cancer, Gastric cancer
Spartalizumab	PD‐1	BRAF mutated maligant melanoma
Cemiplimab	PD‐1	Squamous cell skin cancer
Atezolizumab	PD‐L1	Breast cancer, Non‐small cell lung cancer, Small cell lung cancer
Avelumab	PD‐L1	Merkel cell cancer, Renal cell carcinoma
Durvalumab	PD‐L1	Non‐small cell lung cancer

Although therapies that activate the immune system against cancer cells have been studied for a long time, the use of the patient's own immune system for cancer treatment was not established. Recently, the effectiveness of both immune checkpoint inhibition therapy and chimeric antigen receptor (CAR)‐T cell therapy has proved to be promising.^39^, ^40^ Immunotherapy has moved to the forefront of cancer treatment strategies.

There are two major reasons why proving the efficacy of cancer immunotherapies was difficult for some time. First, cancer immunity is strongly suppressed. Signal transduction from immune checkpoint compounds, such as PD‐1 and CTLA4, strongly inhibits cytotoxic T cells (CTLs).^38^ This checkpoint mechanism can prevent the immune system from attacking cancer cells. The development of immune checkpoint inhibitors has arisen from the discovery of this mechanism. Inhibition of immune checkpoint molecules with neutralizing antibodies can release the suppression of cancer‐specific CTLs, activate immunity, and promote cancer elimination. The effectiveness of immune checkpoint antibodies has been confirmed and clinically applied to many solid cancers such as melanoma,^41^ lung cancer,^42^ urothelial cancer,^43^ gastric cancer,^44^ and esophageal cancer.^45^ In addition to PD‐1 and CTLA4, new immune checkpoint molecules, such as LAG3, TIGIT, and SIRPA, are also being actively studied.^46^, ^47^, ^48^ Although this therapy is promising, the cancer cases who respond to these therapies are limited. This is because use of this therapy requires the presence of cancer‐specific CTLs in the patient's body. To maximize the therapeutic effect, it is desirable to select appropriate cases and develop useful biomarkers.

The second difficulty for immunotherapy is that T cells do not recognize specific cancer cell antigens and immune accelerators are too weak. One goal of CAR‐T cell therapy is to strengthen the immune accelerator by administering CTLs to the patient's body that recognize specific cancer cell‐specific antigens. A CAR is prepared by fusing a single chain Fv (scFv), derived from a monoclonal antibody that recognizes a specific antigen expressed by cancer cells, with CD3z and costimulatory molecules (CD28, 4‐1BB, and others). Next, the CAR is introduced to the T cells obtained from a cancer patient and CAR‐T cells are made. CAR‐T cells recognize the specific antigen of the cancer cells and are activated to damage these cells. CAR‐T cells recognize cancer‐specific antigens with high antibody specificity and attack the respective cancer cells with strong cytotoxic activity and high proliferative activity. CAR‐T therapy is effective in blood cancers such as B‐cell acute lymphoblastic leukemia and myeloma.^49^, ^50^ While CAR‐T cell therapy has a high therapeutic effect, a frequent and serious adverse event called cytokine release syndrome has been observed in some patients.^51^, ^52^ The development of a technique for suppressing the occurrence of cytokine release syndrome is anticipated. In addition, the development of CAR‐T cell therapies for solid tumors is ongoing.

Recently, there was new progress made in treating gastrointestinal cancer patients. For MSI‐H colorectal cancer, the combination therapy with nivolumab and ipilimumab was approved. From the nivolumab plus ipilimumab cohort of CheckMate‐142, progression‐free survival rates were 76% (9 months) and 71% (12 months); respective overall survival rates were 87% and 85% which were quite high. This new treatment will benefit MSI‐H colorectal cancer patients.^53^

Thus, it is expected that further understanding of cancer immune mechanisms and the development of various immunotherapies will contribute to great progress in cancer treatment.

One problem for immunotherapy is that there is no certain predictive biomarker. It was thought that the expression of PD‐1 or PD‐L1 would predict the effect. However, this was not the case. To find a new biomarker, we assessed the cytolytic activity (CYT) score. The CYT score is a new index of cancer immunity calculated from the mRNA expression levels of GZMA and PRF1. We are now evaluating CYT score in gastric cancer patients (data not published). The development in the biomarker search will benefit many gastrointestinal cancer patients.

## 5 ADVANTAGES FOR USING ORGANOIDS IN CANCER RESEARCH

The three‐dimensional (3D) organoid system is a cell culture‐based, novel, and physiologically relevant biologic platform.^54^ An organoid is a miniaturized and simplified version of an organ that is produced in vitro in 3D and shows realistic microanatomy. With only one to a few cells isolated from tissue or cultured cells as the starting material, organoids are grown and passaged in a basement membrane matrix, which contributes to their self‐renewal and differentiation capacities.^54^, ^55^ The technique used for growing organoids has rapidly improved since the early 2010s with the advent of the field of stem cell biology. The characteristics of stem, embryonic stem cells (ES cells), or induced pluripotent stem cells (iPS cells) that allow them to form an organoid in vitro are also found in multiple types of carcinoma tissues and cells. Therefore, cancer researchers have applied ES cells or iPS cells in their field.^56^, ^57^, ^58^

Organoid formation generally requires culturing stem cells or their progenitor cells in 3D.^54^, ^55^ The morphological and functional characteristics of various types of carcinoma tissue have been recapitulated in organoids that were generated from single‐cell suspensions or cell aggregates. These suspensions or aggregates were isolated from murine and human tissues or cultured cells, as well as from cancer stem cells propagated in culture. The structures of the organoids show the potential of cancer stem cell self‐renewal, proliferation, and differentiation abilities, and also provide insights into the roles of molecular pathways and niche factors that are essential in cancer tissues.^56^, ^57^, ^59^, ^60^, ^61^, ^62^ The organoid system also has been utilized for studying multiple biological processes, including motility, stress response, cell‐cell communications, and cellular interactions that involve a variety of cell types such as fibroblasts, endothelial cells, and inflammatory cells. These interactions are mediated via cell surface molecules, extracellular matrix proteins, and receptors in the microenvironment under homeostatic and pathologic conditions.

Although the organoid system is a complex and not effortless procedure that requires specific media, supplements, and many tricky techniques,^58^, ^63^ application of this system has been extended to a variety of cell types from different carcinomas (colorectal, pancreatic, prostate, breast, ovary, and esophageal cancers).^56^, ^57^, ^59^, ^60^, ^61^ An organoid is generally induced within a few days to weeks, and is faster and less costly than the murine xenograft assay. Furthermore, applying novel genetic manipulations (e.g. CRISPR‐Cas9) can be carried out in the organoid system.^64^, ^65^

Kasagi et al modified keratinocyte serum‐free medium to grow 3D organoids from endoscopic esophageal biopsies, immortalized human esophageal epithelial cells, and murine esophagi. Esophageal 3D organoids serve as a novel platform to investigate regulatory mechanisms in squamous epithelial homeostasis in the context of esophageal cancers.^64^

We anticipate that many experimental results that utilize the organoid system will be published in the future.

The 3D organoid system has emerged in the past several years as a robust tool in basic research with the potential to be used for personalized medicine.^66^ By passaging dissociated primary structures to generate secondary 3D organoids, this system can be performed using live tissue pieces obtained from biopsies, operative‐resected specimens, or even frozen tissues. This method has the potential to transform personalized therapy. For example, in the case of cancer recurrence, an effective chemotherapy can be selected by testing the chemotherapeutic sensitivity of cancer‐derived organoids from an individual patient's tissue stocks. In many cases, a patient's organoid accumulation is helpful for testing the sensitivity of novel therapeutic agents for treating carcinoma.^66^ Hence, it appears that organoid biology will further develop with a goal of translating the research into personalized therapy.

## 6 SUMMARY AND FUTURE DIRECTIONS

**FIGURE 1:**
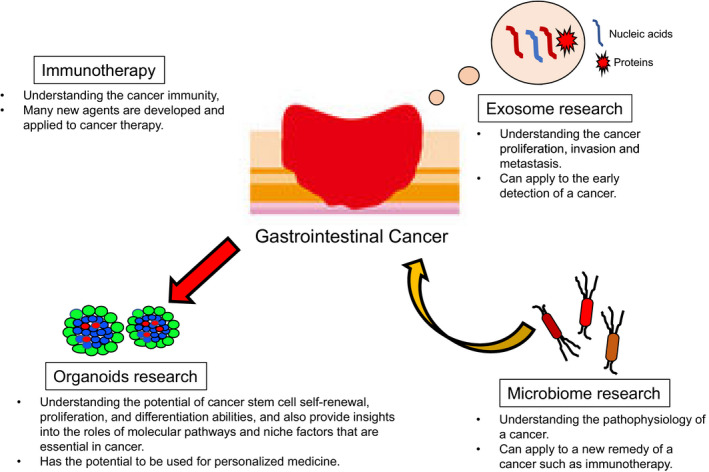
The summary of the four cancer research areas. In this figure the summary of the four cancer research areas is shown: exosome, microbiome, immunotherapy, and organoid research

This review describes four new cancer‐related studies: exosomes, microbiome, immunotherapy, and organoids (Figure 1).

Since exosomes are released in blood or urine, if the capturing system is established, it will be a less invasive test to diagnose cancer. In the present, the presence of circulating tumor DNA (ctDNA) is one of the tools to detect the minimal residual disease. However, since ctDNA is only DNA, it is difficult to spread to cancer research. In that respect, as exosomes include not only DNA but also other nucleic acids and proteins, this will be a new tool for cancer research such as the diagnosis of early cancer.

Microbiome may lead to improved cancer diagnosis and treatment. Detecting a specific microbiome in a gastrointestinal tract may predict a specific cancer. And changing microbiome in some way may result in preventing cancer development.

Organoids may help address the problem of drug resistance, and also lead to the development of personalized therapy. However, producing organoids takes time and testing the drug resistance may take more time. If we could overcome these problems, the research into organoids can contribute to overcoming cancer.

As shown in Table 3, many new studies and findings are reported into this field of research. These four novel cancer research areas will make many contributions to the diagnosis and treatment of cancer.

**TABLE 3:** Recent studies on exosome, microbiome, immunotherapy, and organoids

Research	Author	Recent studies in gastrointestinal cancers	Journal
Exosome	Liu et al	Serum exosomal miR‐766‐3p could serve as a prognostic marker for the assessment of esophageal squamous cell carcinoma.	*Cancer Sci*. 111(10):3881‐92, 2020
	Lin et al	Salivary exosomal GOLM1‐NAA35 chimeric RNA (seG‐NchiRNA) in esophageal squamous cell carcinoma constitutes an effective candidate noninvasive biomarker for the convenient, reliable assessment of therapeutic response, recurrence, and early detection.	*Clin Cancer Res*. 25(10):3035‐45, 2019
	Liu et al	MiR‐128‐3p delivery via exosomes may be a promising diagnostic and prognostic marker for oxaliplatin‐based chemotherapy for colorectal cancer	*Mol Cancer*. 18(1):43, 2019
	Lan et al	MiRNA‐containing exosomes derived from M2 macrophages regulate migration and invasion of colorectal cancer cells.	*Cancer Res*. 79(1):146‐58, 2019
	Bernard V et al	Longitudinal monitoring using liquid biopsy samples through exosomal DNA and ctDNA provides both predictive and prognostic information relevant to therapeutic stratification in pancreatic cancer.	*Gastroenterology*. 156(1):108‐18, 2019
Microbiome	Roberti et al	The ileal microbiota dictates tolerogenic versus immunogenic cell death of ileal intestinal epithelial cells (IECs) and the accumulation of TFH cells in patients with CC	*Nat Med*. 26(6):919‐31, 2020
	Mage et al	This study identifies a previously unknown microbial metabolite immune pathway activated by immunotherapy that may be exploited to develop microbial‐based adjuvant therapies.	*Science*. 369(6510):1481‐9, 2020
	Manzano et al	This study describes a distinct mutational signature in colorectal cancer and implies that the underlying mutational process results directly from past exposure to bacteria carrying the colibactin‐producing pks pathogenicity island.	*Nature*. 580(7802):269‐73, 2020
	Gu et al	CEACAM proteins disrupt TGFB signaling, which alters the composition of the intestinal microbiome to promote colorectal carcinogenesis.	*Gastroenterology*. 158(1):238‐52, 2020
	Song et al	The features of the intestinal microbiome might be used for CRC screening and modified for chemoprevention and treatment.	*Gastroenterology*. 158(2):322‐40, 2020
Immunotherapy	Le DT et al	Pembrolizumab is effective with a manageable safety profile in patients with MSI‐H/dMMR colorectal cancer (KEYNOTE‐164).	*J Clin Oncol*. 38(1):11‐9, 2020
	Kojima et al	Pembrolizumab prolonged OS vs chemotherapy as second‐line therapy for advanced esophageal cancer in patients with PD‐L1 CPS ≥ 10, with fewer treatment‐related adverse events (KEYNOTE‐181).	*J Clin Oncol*. 38(35):4138‐48, 2020
	Hack et al	IMbrave 050: a Phase III trial of atezolizumab plus bevacizumab in high‐risk hepatocellular carcinoma after curative resection or ablation	*Future Oncol*. 16(15):975‐89, 2020
	Kato et al	Nivolumab was associated with a significant improvement in overall survival and a favorable safety profile compared with chemotherapy in previously treated patients with advanced oesophageal squamous cell carcinoma, and might represent a new standard second‐line treatment option for these patients (ATTRACTION‐3).	*Lancet Oncol*. 20:1506‐17, 2019
	Overman et al	Nivolumab plus ipilimumab demonstrated high response rates, encouraging progression‐free survival and OS at 12 mo, manageable safety, and meaningful improvements in patients with MSI‐H/dMMR colorectal cancer (CheckMate‐142)	*J Clin Oncol*. 36(8):773‐9, 2018
	Kang et al	In ATTRACTION‐2 study, the survival benefits indicate that nivolumab might be a new treatment option for heavily pretreated patients with advanced gastric or gastro‐oesophageal junction cancer.	*Lancet*. 390(10111):2461‐71, 2017
Organoids	Yao et al	The patient‐derived organoids predict locally advanced rectal cancer patient responses in the clinic and may represent a companion diagnostic tool in rectal cancer treatment.	*Cell Stem Cell*. 26(1):17‐26, 2020
	Kong et al	This study presents a method to predict cancer patient drug responses using pharmacogenomic data derived from organoid models by combining the application of gene modules and network‐based approaches.	*Nat Commun*. 11(1):5485, 2020
	Bruun et al	Variation in drug sensitivities was reflected at the transcriptomic level in the patient‐derived organoids from multiple colorectal cancer liver metastases, suggesting potential to develop gene expression‐based predictive signatures to guide experimental therapies.	*Clin Cancer Res*. 26(15):4107‐19, 2020
	Ganesh et al	The biology and drug sensitivity of RC clinical isolates can be efficiently interrogated using an organoid‐based, ex vivo platform coupled with in vivo endoluminal propagation in animals.	*Nat Med*. 25(10):1607‐14, 2019

## DISCLOSURE

Conflict of Interest: All the authors have no conflict of interest to disclose.
